# Divergence exists in the subcellular distribution of intramuscular triglyceride in human skeletal muscle dependent on the choice of lipid dye

**DOI:** 10.1007/s00418-020-01898-2

**Published:** 2020-07-05

**Authors:** Juliette A. Strauss, Daisy A. Shepherd, Myfanwy Macey, Emily F. P. Jevons, Sam O. Shepherd

**Affiliations:** 1 Research Institute for Sport and Exercise Science, Liverpool John Moores University, Liverpool, L3 3AF UK; 2 Clinical Epidemiology and Biostatistics Unit, Murdoch Children’s Research Institute, Royal Children’s Hospital, Victoria, 3052 Australia; 3 Department of Paediatrics, The University of Melbourne, Victoria, 3010 Australia

**Keywords:** BODIPY, Oil Red O, Intramuscular triglyceride, Skeletal muscle

## Abstract

Despite over 50 years of research, a comprehensive understanding of how intramuscular triglyceride (IMTG) is stored in skeletal muscle and its contribution as a fuel during exercise is lacking. Immunohistochemical techniques provide information on IMTG content and lipid droplet (LD) morphology on a fibre type and subcellular-specific basis, and the lipid dye Oil Red O (ORO) is commonly used to achieve this. BODIPY 493/503 (BODIPY) is an alternative lipid dye with lower background staining and narrower emission spectra. Here we provide the first quantitative comparison of BODIPY and ORO for investigating exercise-induced changes in IMTG content and LD morphology on a fibre type and subcellular-specific basis. Estimates of IMTG content were greater when using BODIPY, which was predominantly due to BODIPY detecting a larger number of LDs, compared to ORO. The subcellular distribution of intramuscular lipid was also dependent on the lipid dye used; ORO detects a greater proportion of IMTG in the periphery (5 μm below cell membrane) of the fibre, whereas IMTG content was higher in the central region using BODIPY. In response to 60 min moderate-intensity cycling exercise, IMTG content was reduced in both the peripheral (− 24%) and central region (− 29%) of type I fibres (*P *< 0.05) using BODIPY, whereas using ORO, IMTG content was only reduced in the peripheral region of type I fibres (− 31%; *P *< 0.05). As well as highlighting some methodological considerations herein, our investigation demonstrates that important differences exist between BODIPY and ORO for detecting and quantifying IMTG on a fibre type and subcellular-specific basis.

## Introduction

Although lipid is predominantly stored in adipose tissue in humans, a small amount (~ 200 g) is also stored in skeletal muscle in the form of intramuscular triglyceride (IMTG). Healthy, trained individuals have large IMTG stores that are located in close proximity to, and even in contact with, mitochondria (Hoppeler 1999; Shaw et al. 2008), and IMTG is therefore an important fuel source during moderate-intensity exercise (van Loon 2004). However, obese type 2 diabetes patients also have large IMTG stores but are insulin resistant (Goodpaster et al. 2001; Pan et al. 1997; van Loon et al. 2004). The fact that athletes are able to combine high IMTG stores with superior insulin sensitivity has been termed the athletes paradox (Goodpaster et al. 2001). Moreover, it is because IMTG contributes minimally as a fuel source during exercise in sedentary, obese individuals and type 2 diabetes patients (van Loon 2004), that leads to IMTG accumulation and the development of insulin resistance (Chalkley et al. 1998; Griffin et al. 1999; Itani et al. 2002; Yu et al. 2002). Given the continued rise in obesity and type 2 diabetes prevalence, there is a sustained effort to understand the link between lipid storage and metabolism in skeletal muscle and health and disease.

The first studies to investigate IMTG use during exercise employed biochemical triacylglycerol extraction analyses in mixed muscle samples obtained before and after exercise to determine net changes in IMTG content. The findings from these studies, however, were equivocal with some reporting a decrease in IMTG following exercise (Essen-Gustavsson and Tesch 1990; Essen et al. 1977; Hurley et al. 1986; Watt et al. 2002), whereas others found no difference in IMTG content (Bergman et al. 1999; Kiens et al. 1993; Kiens and Richter 1998; Starling et al. 1997). The discrepant findings can be attributed to a large between-biopsy variability, driven by the presence of extramyocellular lipids which are located between the muscle fibres but which cannot be excluded from analysis of mixed muscle homogenates (van Loon 2004). Using immunofluorescence microscopy in conjunction with a lipid dye removes the confounding effect of extramyocellular lipids and permits IMTG content to be determined in a fibre type-specific manner (Koopman et al. 2001). Oil red O (ORO) is the most commonly used lipid dye to investigate lipid metabolism in skeletal muscle, and has been employed to demonstrate that IMTG content and use during exercise is greatest in type I fibres (Shepherd et al. 2013; van Loon et al. 2003). Importantly though, ORO does not exclusively stain IMTG, but labels all neutral lipids (e.g., phospholipids within membranes). Furthermore, ORO has a large emission spectrum (576–750 nm) which crosses the 543 nm, 594 nm and 633 nm excitation/emission channels (other commonly used colour channels in immunofluorescence microscopy), making some co-staining combinations difficult and also resulting in a high background level of staining. The use of ORO to investigate lipid droplet (LD) morphology may also be inappropriate, since ORO is often dependent on the use of isopropanol, which has been reported to induce artificial fusion of LD in cultured mammalian cells (Listenberger and Brown 2007). An alternative neutral lipid stain is BODIPY 493/503 (BODIPY), which emits a bright green fluorescent signal. Methods for using BODIPY in combination with confocal immunofluorescence microscopy have been demonstrated in vitro (Listenberger et al. 2016), in rodent skeletal muscle (Prats et al. 2006; Spangenburg et al. 2011) and human skeletal muscle (Jevons et al. 2020; Prats et al. 2013; Whytock et al. 2020). Importantly, BODIPY is more specific for detecting IMTG-containing LD’s than Nile red in flow cytometry applications (Gocze and Freeman 1994), and when imaged using fluorescence emitted between 570 and 650 nm, it has been confirmed that most of the fluorescence is due to detection of IMTG (Prats et al. 2013). BODIPY may therefore provide the most accurate tool to identify LDs and quantify IMTG content in skeletal muscle, but a quantitative comparison between BODIPY and ORO is yet to be made.

With this in mind, the overarching aim of the current investigation was to compare BODIPY and ORO for quantifying IMTG content and LD morphology in human skeletal muscle. First, we investigated fibre type and region (peripheral and central)-specific differences in IMTG content detected using BODIPY and ORO in muscle biopsies obtained after an overnight fast (study 1). In this context, subcellular distribution is an important consideration because IMTG use during exercise occurs primarily in the central (intermyofibrillar) region of type I fibres of the cell (Jevons et al. 2020; Koh et al. 2017), and IMTG deposited in the peripheral (subsarcolemmal) region is inversely related to insulin sensitivity (Nielsen et al. 2017; Nielsen et al. 2010). We also critically reviewed several steps in the immunohistochemical protocol and analytical procedure that can affect the visualisation and quantification of IMTG content. Thus, using the optimised protocol and analytical procedure, we aimed to compare BODIPY and ORO for quantifying exercise-induced changes in IMTG content (study 2).

## Methods

### Subject characteristics—studies 1 and 2

The muscle samples used across this investigation were collected as part of two previous studies from our laboratory (Shepherd et al. 2013, 2014). All participants were healthy, lean sedentary males, with selected subject characteristics for both studies presented in Table 1. All participants provided written informed consent, following a verbal explanation of the nature and risks involved in the study (approved by the Black Country NHS Research Ethics Committee, West Midlands, UK).

**Table 1:** Subject characteristics

	Study 1	Study 2
*n*	6	8
Age (years)	20 ± 1	23 ± 3
Height (m)	1.69 ± 0.06	1.75 ± 0.06
Body Mass (kg)	70.3 ± 7.3	71.9 ± 6.5
BMI (kg.m^−2^)	24.8 ± 3.0	23.5 ± 1.9
VO_2peak_ (L.min^−1^)	2.78 ± 0.38	2.96 ± 0.85
VO_2peak_ (ml.min^−1^.kg^−1^)	39.8 ± 5.8	41.1 ± 11.6
*W*_max_ (W)	183 ± 28	218 ± 35

Data presented are mean ± SD

*BMI* body mass index, *W*_*max*_ maximum workload

### Experimental procedures

For studies 1 and 2, maximal oxygen uptake (VO_2max_) was initially determined during a progressive exercise test to exhaustion on an electronically braked cycle ergometer (Lode BV, Groningen, The Netherlands). At least 96 h later, subjects arrived at the laboratory following an overnight fast (> 10 h) and a muscle sample was obtained from the *m. vastus lateralis* of one leg under local anaesthesia (~ 5 ml 1% lidocaine) using the percutaneous needle biopsy technique with suction (Bergstrom et al. 1967). In study 2, participants then undertook 60 min of moderate-intensity exercise on an electronically braked cycle ergometer set at a workload equivalent to 65% VO_2max_. A second muscle biopsy was taken immediately post-exercise from a separate incision made 2 cm proximal to the first on the same leg. All muscle samples were blotted of excess blood and any visible fat and collagen was separated from the muscle tissue and discarded. The biopsy sample was then embedded in Tissue-Tek OCT Compound (Sakura Finetek Europe, Zoeterwoude, The Netherlands) on a cork board which was immediately frozen in liquid nitrogen-cooled isopentane (Sigma-Aldrich, Dorset, UK) and stored in pre-cooled cryotubes at − 80 °C for histological analyses.

### Immunofluorescence staining—Studies 1 and 2

Cryosections (5 µm) were cut using a microtome (Bright Instrument Company Limited, Huntingdon, England) housed within a cryostat at – 25 °C. The sections were collected onto uncoated, ethanol-cleaned glass slides (VWR International Ltd, Leicestershire, UK) and for study 2 sections from pre and post exercise biopsies were placed on the same slide to control for slide to slide variability. Samples were prepared for histological analyses of IMTG in the same way for each lipid dye. The detailed methodology for both BODIPY and ORO can be found below. In preparation, cryosections were fixed for 1 h in 3.7% formaldehyde. Following 3 × 30 s rinses in doubly distilled water (dd H_2_O), the slides were permeabilised in 0.5% Triton X-100 for 5 min. Subsequently, slides were washed 3 × 5 min in phosphate buffered saline (PBS, 137 mM sodium chloride, 3 mM potassium chloride, 8 mM disodium hydrogen phosphate and 3 mM potassium dihydrogen phosphate, pH 7.4). Fibre type was determined using mouse anti-slow twitch myosin heavy chain antibody (MHCI; a4.840c developed by Dr Blau, DSHB, Iowa, USA) to label type I fibres and mouse anti-fast twitch IIA myosin heavy chain antibody (MHCIIa; 2F7, DSHB, Iowa, USA) to label type IIa fibres. Any fibres with no positive signal were then deemed to be type IIx fibres. Rabbit anti-laminin was used to identify the cell membrane (and used as a cell border for the purposes of analysis). The antibody solution was contained on the sections during the incubation through the use of a hydrophobic barrier pen (ImmEdge, Vector Laboratories, Burlingame, USA). Following 3 × 5 min wash in PBS, appropriate secondary antibodies were applied. The secondary antibodies were Alexa Fluor GAMIgG for anti-MHC IIa, Alexa Fluor GAMIgM for anti-MHCI and Alexa Fluor GARIgG for anti-laminin. Following a further 3 × 5 min wash in PBS the sections were incubated in either BODIPY or ORO (specific details of which are outlined below). At the end of the staining procedures coverslips were mounted onto the dried slides using Vectashield (H1000, Vectorlabs) and sealed using nail polish.

Oil Red O: A working solution of ORO was made for each staining procedure and consisted of 100 mg ORO (Sigma-Aldrich, UK) in 20 ml 60% triethylphosphate (Sigma-Aldrich, UK). Twelve ml of working solution was added to 8 ml dd H_2_O and filtered twice using Whatman^®^ Grade 1 filter paper (11 µm particle retention) to remove any residual ORO crystals. The filtered ORO solution was then applied to the muscle sections for 30 min before a 10 min wash under cold, slowly running water.

BODIPY 493/503: A working solution of BODIPY (Difluoro{2-[1-(3,5-dimethyl-2*H*-pyrrol-2-ylidene-*N*)ethyl]-3,5-dimethyl-1*H*-pyrrolato-*N*}boron) 493/503 was diluted from the stock solution as supplied (Sigma Aldrich, UK). The stock solution was diluted in PBS at a ratio of 1:50 to create a working solution. BODIPY was applied to the muscle samples for 20 min before a 5 min wash in PBS. Due to the light sensitive nature of BODIPY, all work with BODIPY including the final washes and mounting of coverslips was completed in a dark room.

### Image capture, processing and data analysis

Images of cross-sectional orientated sections stained for ORO and BODIPY were captured using an inverted confocal microscope (Zeiss LSM710; Oberkochen, Germany) with a 63 × (1.4 NA) oil immersion objective. The pinhole was set at 1AU for all imaging to avoid pixel saturation. Images were captured at a 1.1 zoom allowing for one complete cell to fill the field of view. ORO was excited with the HeNe 543 nm laser whilst BODIPY was excited using the 488 nm argon laser. Alexa Fluor 633 was excited with the HeNe 633 nm laser and detected with 638–747 nm emission. Alexa Fluor 543 was excited with 543 nm laser and detected with 548–623 nm emission. Alexa Fluor 405 was excited using the 405 nm line of the diode laser and detected with 371–422 nm emission. BODIPY emitted fluorescence was detected from 570–650 nm to specifically detect IMTG as suggested in Prats et al. (2013). The images were acquired at a resolution of 1024 × 1024 pixels and stored in 24‐bit tagged image format file format. Identical settings were used for all image capture for each fluorescent tag/dye within each participant.

As part of study 1, we aimed to investigate the variability and photostability of ORO and BODIPY staining. The coefficient of variation (CV) of the BODIPY and ORO staining procedures was determined through the sequential imaging of serial cryosections on one slide. The same five type I fibres were identified on five serial cryosections and images were captured at a 1.1 × zoom allowing for ~ 1 cell to fill the field of view. The image capture settings were identical to those listed above. To determine the photostability of the two dyes, the same five type I muscle fibres were imaged and total IMTG content was quantified from images captured 1, 3, 6, 8, and 10 days following the initial staining. Slides were stored in the dark and at room temperature between imaging.

**Fig. 1:**
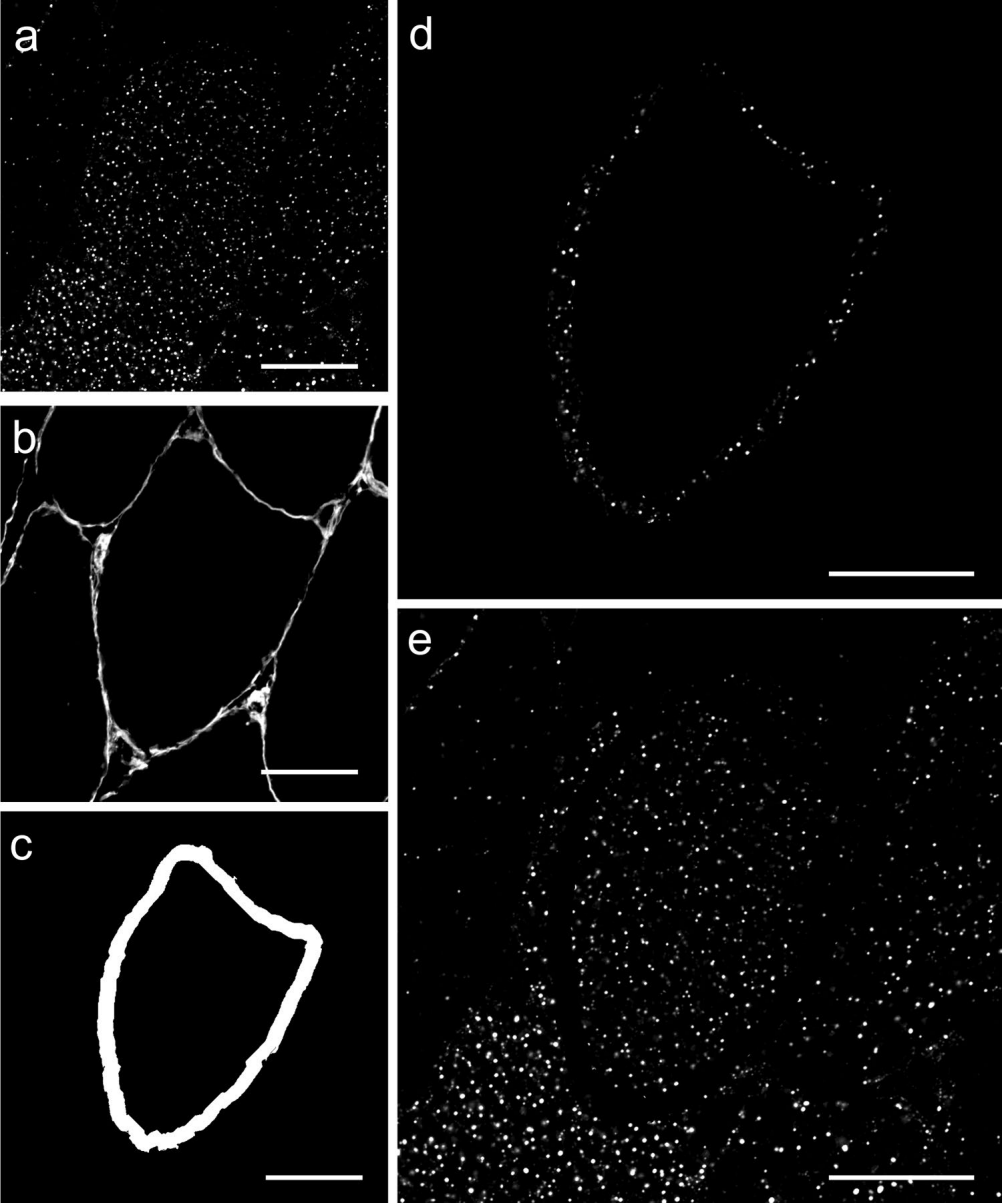
Region-specific image analysis. A grey scale image was created of both BODIPY staining (**a**) and the cell membrane (labelled using anti-laminin) (**b**). A 5 µm region was identified within the inside of the membrane (**c**). LD were then identified in this 5 µm (peripheral) region (**d**) in addition to the remainder of the cell (central region) (**e**). The same procedure was used for region-specific analysis of images of ORO staining. White bar = 30 µm

Confocal images were processed using Image-Pro Plus 5.1 software (Media Cybernetics, MD, USA). To investigate the fibre type distribution of IMTG an intensity threshold was uniformly selected for both BODIPY and ORO to represent the positive IMTG signal. The corresponding laminin image was used to create a binary (black and white) image of the cell border and therefore the outline of the cell. A region-specific (peripheral vs. central) analysis was performed; the cell border was then used to create a 5 µm ring inside the cell border, and was defined as the peripheral region of the cell. The remaining area was defined as the central region of the cell (Fig. 1). IMTG content was calculated for the whole cell and both the peripheral and central regions and in all cases expressed as the positively stained area fraction relative to the total area of interest (i.e., area of the cell defined as the peripheral or central region or the total cell). The number of LDs was expressed relative to the area of interest. Data on the area (size; µm^2^) of each individual spot (LD) were collected per image, and used to calculate 1) the frequency of LDs over a range of sizes (100 nm groups), and 2) the mean area of individual LDs in an area of interest. For study 1, for each lipid dye analysis was completed for 20 type I fibres, 16 (range 15–19) type IIa fibres and 9 (range 6–12) type IIx fibres were analysed per participant (total of 520 fibres across all participants; 262 for ORO and 258 for BODIPY). For ORO analysis this resulted in 5117 ± 681 LDs being analysed per participant (3237 ± 470 type I fibres, 1480 ± 216 type IIa fibres and 365 ± 41 type IIx fibres). For BODIPY analysis, this resulted in a mean of 12695 ± 2138 LDs being analysed per participant (7799 ± 3267 type I fibres, 3936 ± 2164 type IIa fibres and 960 ± 433 type IIx fibres). For study 2, 13 type I, 13 type IIa fibres and 8 (range 4–13) type IIx fibres were analysed per time-point (pre or post-exercise) per participant (total of 808 fibres across all participants; 401 for ORO and 407 for BODIPY). For BODIPY, this resulted in a total of 19973 ± 3370 LDs being analysed per participant (12788 ± 7232 type I fibres, 6390 ± 3327 type IIa fibres and 794 ± 433 type IIx fibres). For ORO, a total of 10433 ± 1759 LDs were analysed per participant (6419 ± 3799 type I fibres, 3571 ± 2269 type IIa fibres and 443 ± 253 type IIx fibres).

### Statistics

All data are expressed as mean ± S.D. unless otherwise stated, and the significance threshold was set at *P *< 0.05. For study 1, a three-factor within-subjects repeated measures ANOVA was used to identify fibre type-specific differences in IMTG content, LD size and number between BODIPY and ORO staining based on the analysis of the whole cell, with the factors ‘fibre’ (type I vs. type IIa vs. type IIx fibres), ‘stain’ (BODIPY vs ORO) and ‘region’ (peripheral vs. central). For study 2, exercise-induced changes in IMTG content, LD size and LD number using BODIPY and ORO were examined using a four-factor within-subjects repeated measures ANOVA with the additional factor ‘time’ (pre vs. post-exercise). Significant main effects or interactions were examined using Bonferroni adjustment post hoc analysis. In addition to the above analyses, a resampling without replacement approach was used to explore the stability of estimates as the sample size (i.e., number of fibres sampled) changed. Further detail of this approach is provided in the results section alongside a discussion of the findings.

## Results

### Study 1

#### BODIPY detects more IMTG than ORO

**Fig. 2:**
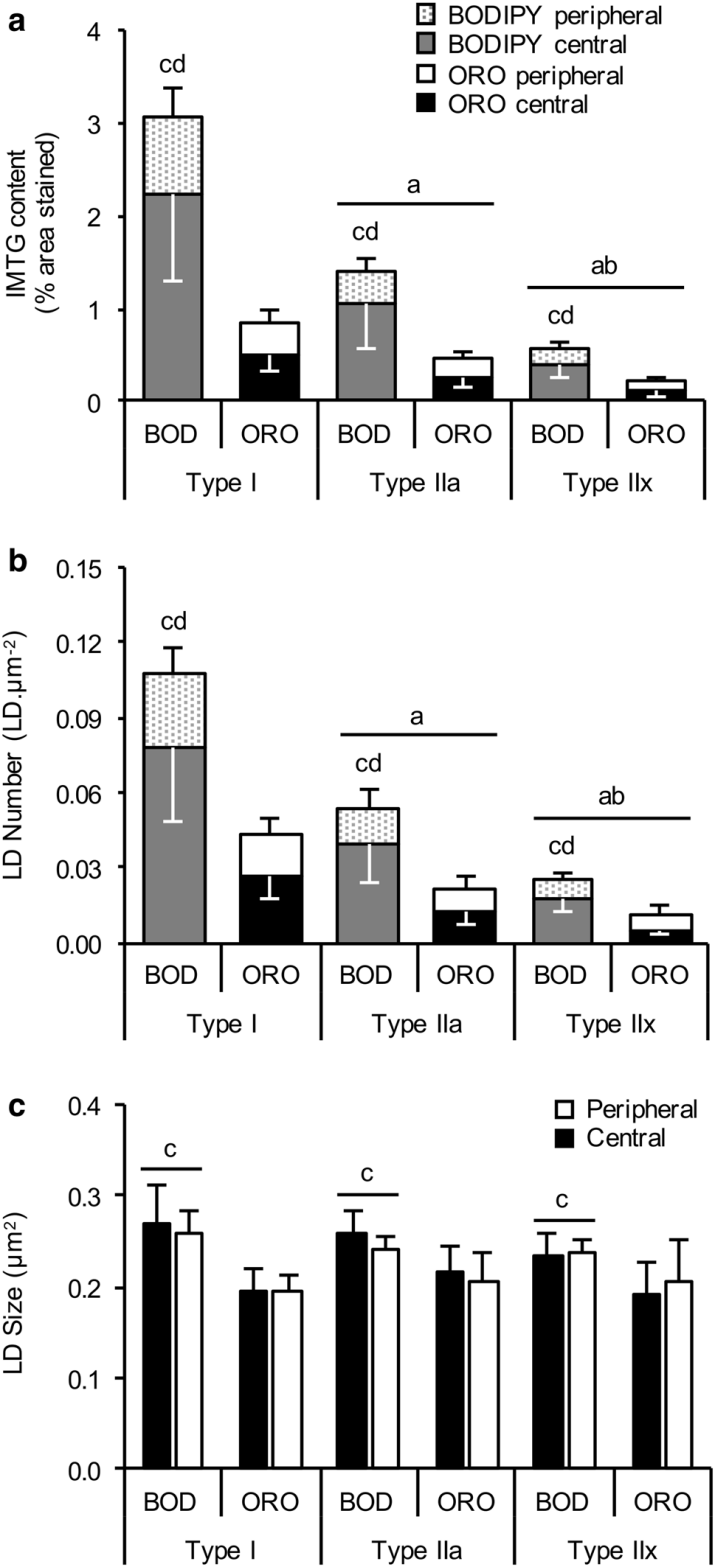
BODIPY detects more lipid than ORO. IMTG content (**a**), LD number (**b**) and LD size (**c**) in peripheral and central subcellular regions in type I, type IIa and type IIx fibres stained using BODIPY and ORO. IMTG content and LD number in each region was normalized to total cell area. ^a^Different from type I fibres (*P *< 0.01), ^b^Different from type IIa fibres (*P *< 0.01); ^c^Different to ORO within fibre type (*P *< 0.05); ^d^Difference between peripheral and central regions (*P *< 0.05). Values are mean ± S.D

Irrespective of the lipid stain used, IMTG content (expressed as  % area stained) followed a hierarchical distribution across fibre types, such that IMTG content in type I fibres > type IIa fibres > type IIx fibres (main effect of fibre type; *P *< 0.01; see Fig. 2a). This fibre type distribution also remained true when IMTG content was determined in both the peripheral (5 µm band beneath the cell membrane) and central region of the cell (main effect of fibre type; *P *< 0.01; see Fig. 2a).

**Fig. 3:**
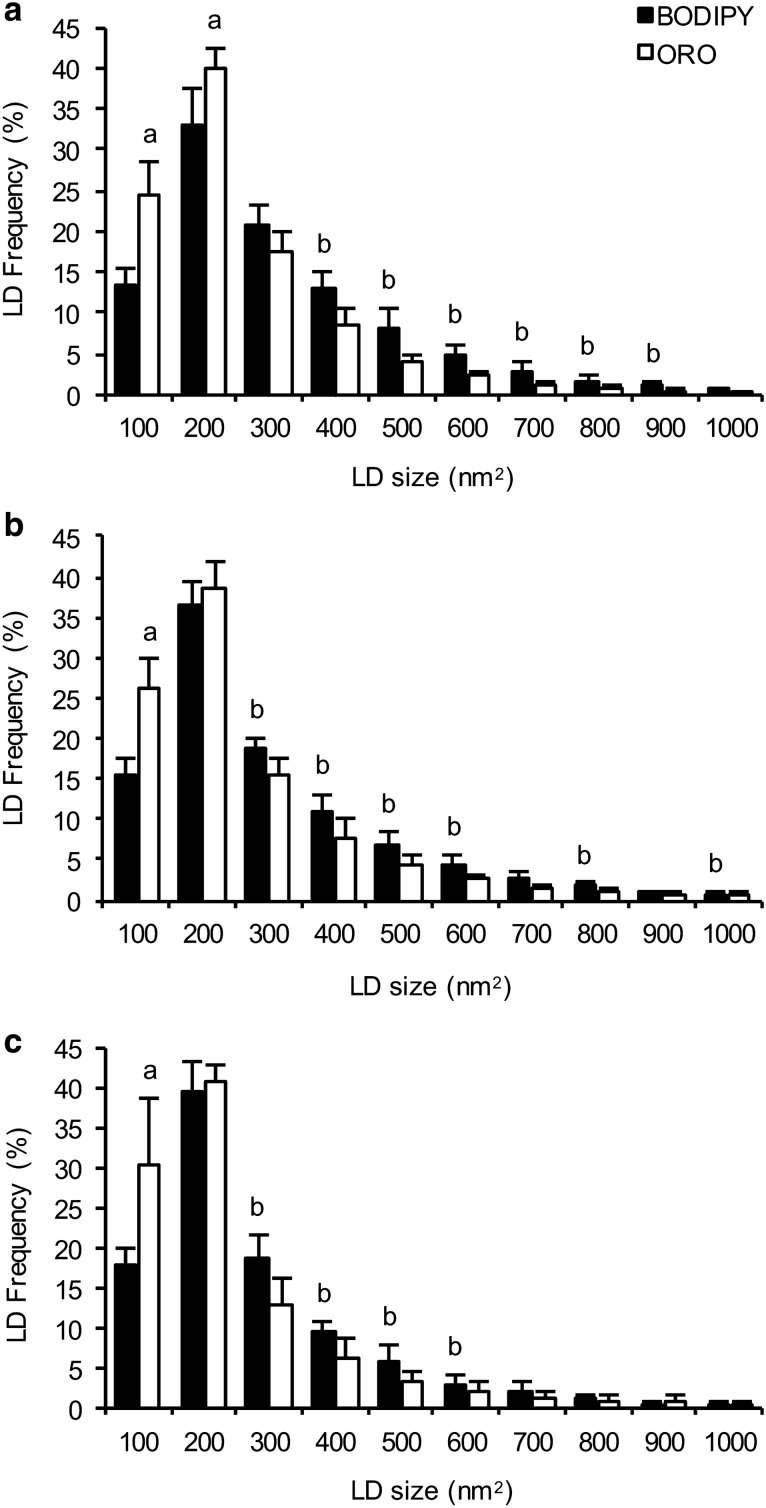
Differences in LD frequency detected using BODIPY and ORO. We interrogated the difference in LD size and number between the two lipid stains by determining the frequency of LDs detected using BODIPY and ORO over a range of LD sizes in type I (**a**), type IIa (**b**) and type IIx (**c**) fibres. ^a^Greater than corresponding BODIPY value (*P *< 0.05); ^b^Greater than corresponding ORO value (*P *< 0.05). Values are mean ± S.D

In all fibre types, IMTG content was greater when BODIPY was used to stain lipid compared to ORO (main effect of stain; *P *= 0.008). Overall, BODIPY detected more LDs than ORO (*P *= 0.03; Fig. 2b), and with a greater mean LD size than ORO (*P *= 0.03; Fig. 2c). We further interrogated the difference in LD size between the two lipid stains by determining the frequency of LDs detected using BODIPY and ORO over a range of LD sizes (Fig. 3). Using this approach, it became apparent that ORO detects a greater number of small LDs (as a proportion of total LDs) compared to BODIPY in type I fibres (LDs up to 200 nm^2^; *P *< 0.01), type IIa fibres and type IIx fibres (LDs up to 100 nm^2^; *P *< 0.01). In contrast, BODIPY detected more LDs compared to ORO that were ≥ 400 nm^2^ in type I fibres (*P *< 0.01), and ≥ 300 nm^2^ in type IIa and type IIx fibres (*P *< 0.01).

#### Subcellular LD distribution differs by lipid dye

When separating the cell into peripheral (5 µm beneath the cell border) and central regions (remainder of the cell), the subcellular distribution of LDs differed depending on whether BODIPY or ORO was used as the lipid stain. In this regard, a significant stain × region interaction was detected (*P *= 0.001), and when this was interrogated the post hoc analysis revealed that IMTG content was greater in the central compared to the peripheral region across all fibre types when using BODIPY (*P *= 0.002; Fig. 2a). However, when using ORO, IMTG content did not differ between the peripheral compared to the central region in each of the fibre types examined (*P *= 0.11). Thus, when calculating the relative distribution of IMTG in each region and fibre type, differences between BODIPY and ORO were also apparent (Table 2). With BODIPY, IMTG content in the peripheral region contributed ~ 28% to total IMTG content across all fibres, which was significantly less than the contribution of the peripheral region (~ 44%) to total IMTG content when analysed using ORO (*P *= 0.01; Table 2). Consequently, IMTG content in the central region contributed significantly more to total IMTG content when detected using BODIPY compared to ORO (*P *= 0.01; Table 2). Whilst the relative distribution of IMTG in each region was uniform across all fibre types using BODIPY, this was not the case for ORO. Using ORO, the proportion of IMTG in the peripheral region compared to the central region was lower in type IIa fibres (*P *= 0.02) and tended to be lower in type I fibres (*P *= 0.07), but was not different between regions in type IIx fibres (*P *= 0.63; Table 2).

**Table 2:** The relative distribution of IMTG at two subcellular locations in human skeletal muscle using Bodipy 493/503 and ORO

Fibre type	Bodipy 493/503		ORO	
	Peripheral	Central	Peripheral	Central
Type I	27 (24–30)^ab^	73 (70–76)^b^	38 (24–52)	62 (48–76)
Type IIa	26 (25–27)^ab^	74 (73–75)^b^	43 (37–48)^a^	58 (52–63)
Type IIx	32 (28–37)^ab^	68 (63–72)^b^	52 (42–62)	48 (38–58)

Values are geometric means and 95% CI

^a^*P *< 0.05 vs. central region within lipid dye

^b^*P *< 0.05 vs. corresponding region detected using ORO

Finally, the regional differences in IMTG content for BODIPY and ORO were entirely attributed to differences in LD number (main effect for region, *P* = 0.039; Fig. 2b) but not LD size (no main effect of region, *P* = 0.492; Fig. 2c).

#### Reproducibility and photostability of BODIPY and ORO

Immunofluorescence analysis of IMTG content was performed in five repeated slides to assess the reproducibility of the BODIPY and ORO. The reproducibility data are displayed in Table 3. The CV for IMTG content was 5.9% with BODIPY and 9.3% with ORO. For BODIPY, the variation in IMTG content could be explained by the variation in both LD number (CV = 3.9%) and LD size (CV = 2.9%). In contrast, the variation in IMTG content using ORO was largely due to variation in LD number (CV = 7.9%) rather than LD size (CV = 1.9%).

**Table 3:** Reproducibility of IMTG content measures with BODIPY and ORO

Measure	Stain	Mean (range)	CV (%)
IMTG content (% area stained)	BODIPY	1.95 (1.85-2.12)	5.9
	ORO	0.53 (0.48-0.59)	9.3
LD number (LD µm^−2^)	BODIPY	0.096 (0.091-0.100)	3.9
	ORO	0.039 (0.034-0.042)	7.9
LD size (µm^2^)	BODIPY	0.232 (0.225-0.242)	2.9
	ORO	0.172 (0.170-0.177)	1.7

**Fig. 4:**
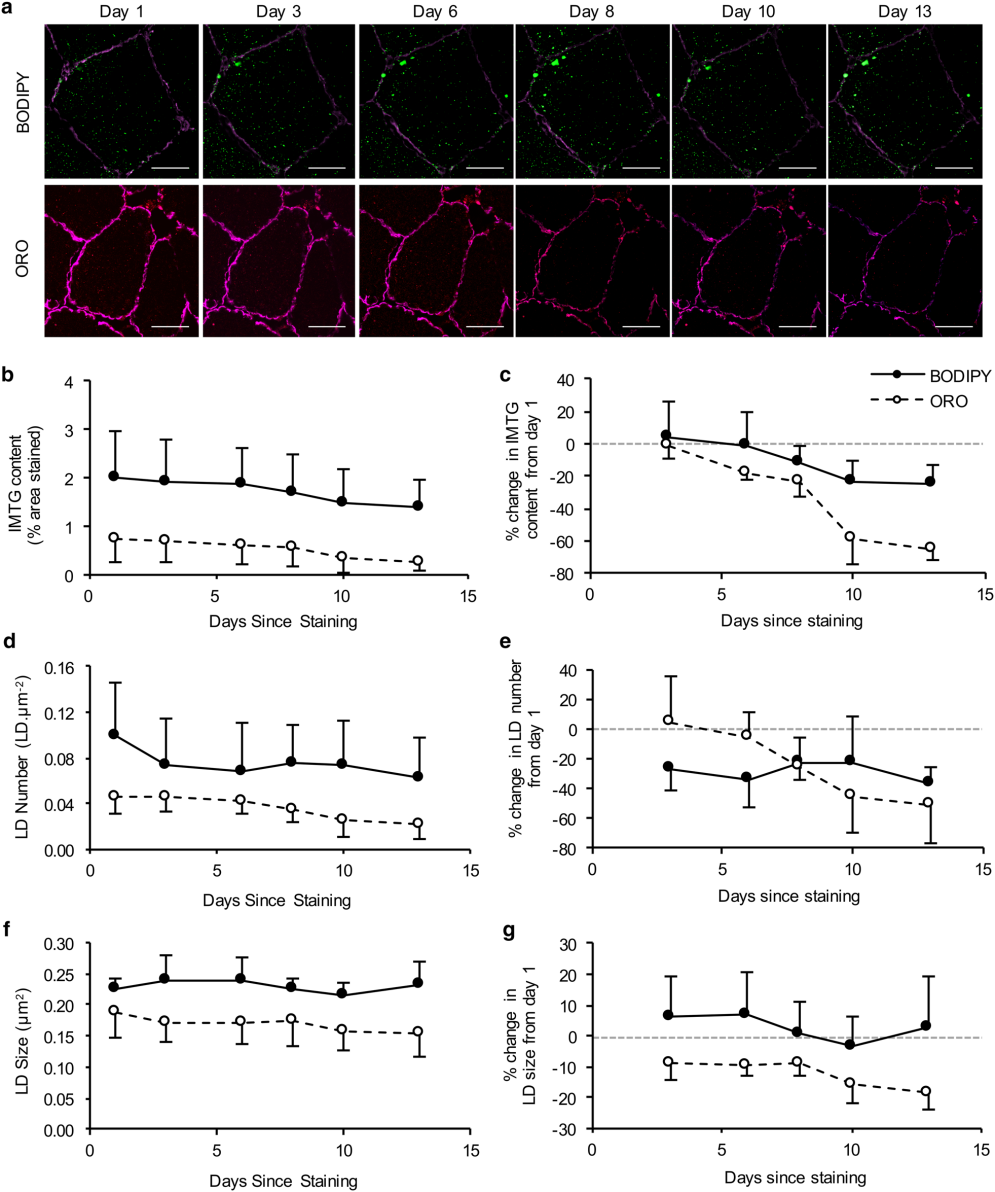
Photo-stability of BODIPY and ORO. **a** Representative images of one type I muscle fibre stained with either BODIPY (green) or ORO (red) and imaged over a 13-day time-course using the same acquisition settings. The cell membrane (laminin) is depicted in pink. White bar = 30 µm. **b** IMTG content (mean of 5 fibres), LD number (**d**) and LD size (**f**) were calculated from the imaged fibres. The  % change from day 1 was subsequently calculated and plotted (**c**, **e**, and **g** for IMTG content, LD number, and LD size, respectively). Values are mean ± S.D

**Fig. 5:**
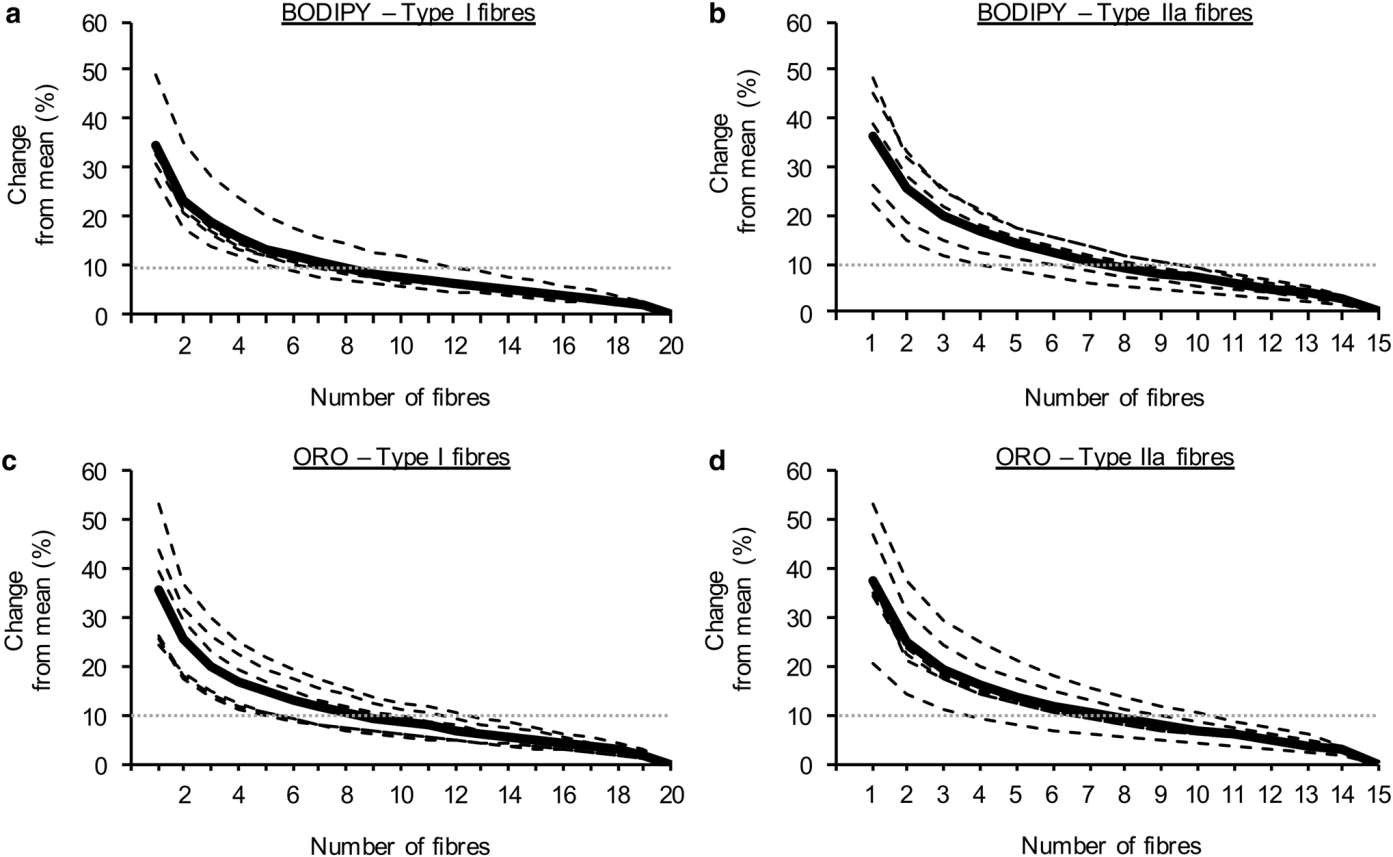
Sample size considerations using BODIPY and ORO. Percentage change from the mean IMTG content obtained depending on the number of fibres analysed using BODIPY and ORO in type I (**a**, **c**) and type IIa fibres (**b**, **d**), respectively. Dashed lines represent individual participant data, whereas the bold continuous line is the average of all participants. Note that the analysis was performed using a maximum of 20 type I fibres and 15 type IIa fibres for each participant. Grey dashed lines represents 10% variation from the mean IMTG content calculated from 20 type I fibres or 15 type IIa fibres

We also performed immunofluorescence analysis of BODIPY and ORO to investigate the photostability of the two lipid dyes. Images were obtained from the same five type I muscle fibres on one occasion (day 1) and then 3, 6, 8, 10 and 13 days following the initial acquisition. After 3 days, IMTG content was similar whether using BODIPY or ORO (Fig. 4b). However, at day 6 there was a divergence, such that IMTG content investigated using ORO was reduced by 19% (compared to day 1), whereas using BODIPY, IMTG content remained unchanged. After day 6, IMTG content decreased irrespective of whether BODIPY or ORO was used to stain lipid (Fig. 5). IMTG content decreased by 32% using BODIPY by day 13, whereas IMTG content was decreased 63% by day 13 when using ORO (Fig. 4). It was interesting to note that using BODIPY, LD number was reduced 28% by day 3, and further reductions after this were relatively small (Fig. 4e). LD size was remained unchanged for BODIPY across the 13-day imaging period. When using ORO, LD number was unchanged at day 6, but then decreased dramatically by 51% by day 13. LD size was slightly reduced at day 3 (− 9%) and remained stable until day 8 using ORO, but was reduced 18% by 13.

#### Sample size considerations using BODIPY and ORO

Due to sampling variability, larger sample sizes produce more reliable results with greater precision and statistical power. Therefore, estimates of IMTG content determined using immunofluorescence microscopy will become more reliable as more fibres in a muscle section are sampled. In this study we aimed to sample 20 type I fibres and 15 type IIa fibres per subject to estimate IMTG content. Collecting this number of images is not only time-consuming when extrapolating to studies with multiple time points and larger sample sizes, but may not always be possible if tissue availability is limited. Therefore, we investigated how reducing the sample size (i.e., number of fibres analysed) would alter the mean IMTG content, compared to quantifying IMTG content from 20 type I or 15 type IIa fibres.

To achieve this, resampling was used to create new realizations of the sampled fibres. This selected a random sample of *n* fibres (without replacement) from the full sample of 20 type I fibres or 15 type IIa fibres. For each random sample, the mean IMTG content across the *n* fibres was calculated. The difference between this sample mean and the overall mean (of 20 or 15 type I or type II fibres, respectively) was determined, to obtain a single estimate of the difference in means. This process was then repeated 10,000 times, each time taking a random sample of *n* cells from the full sample and yielding an estimate of the difference in means. This was iteratively repeated for varying sample sizes (i.e., *n *= 1, 2, …, 20). For each value of *n*, the mean differences from the 10,000 resamples were averaged, to produce a single estimate of the difference between the mean IMTG content for the *n* fibres compared to the mean IMTG content of the full 20 type I fibres (or 15 type IIa fibres). Due to the natural variation associated with an individual participant, the resampling process was applied to each individual’s samples separately.

Figure 5 illustrates how the sample mean deviates from the overall mean for varying sample sizes, expressed as a percentage difference. For the current data, if we sampled at least 13 cells, then the sample mean would be within 10% of the mean of 20 type I fibres and 15 type IIa fibres. This suggests that if we had sampled at least 13 cells, then we could reasonably rely on the sample not being too dissimilar (within 10%) to that of a larger size. Given that in most acute and chronic training interventions the changes expected in IMTG content will exceed 10% (Gemmink et al. 2016; Shepherd et al. 2012, 2013, 2017; van Loon et al. 2003), which also outweighs our reported coefficient of variation for BODIPY or ORO (above), we propose imaging of at least 13 fibres in order to generate a reliable estimate of IMTG content.

### Study 2

#### Choice of lipid stain reveals differences in regional IMTG utilisation during exercise

**Fig. 6:**
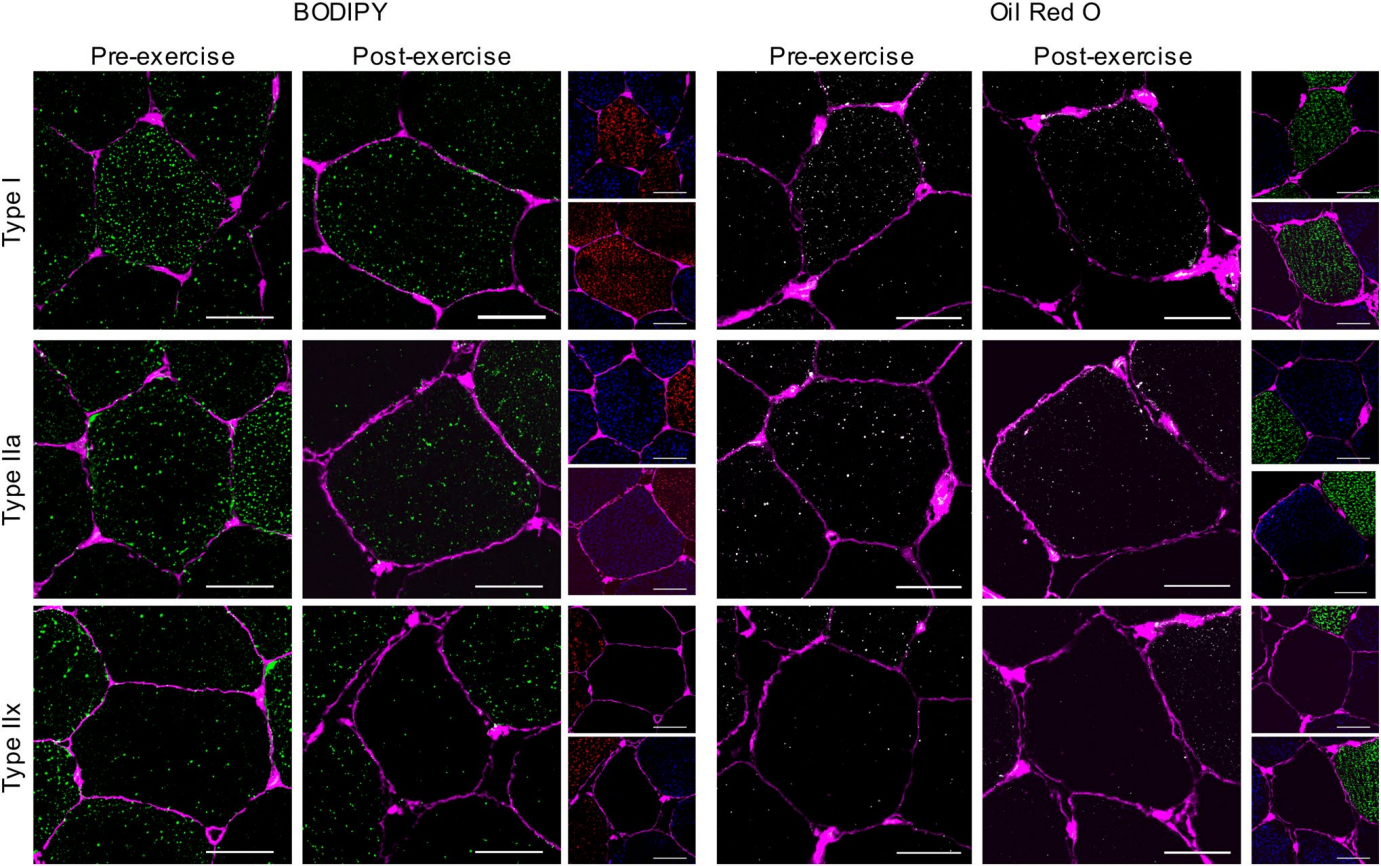
Fibre type specific lipid staining using BODIPY and ORO before and after exercise. For BODIPY panel of images, LDs stained before and after exercise using BODIPY (green) and cell membrane (pink) can be seen in the large images for type I (top), type IIa (middle) and type IIx (bottom). The smaller images show the corresponding fibre type staining with MHCI denoting type I fibres (red) MHCIIa denoting type IIa fibres (blue) and no stain showing type IIx fibres. For ORO panel of images, LDs stained before and after exercise using ORO (greyscale for clarity) and cell membrane (pink) can be seen in the large images for type I (top), type IIa (middle) and type IIx (bottom). The smaller images show the corresponding fibre type staining with MHCI denoting type I fibres (green) MHCIIa denoting type IIa fibres (blue) and no stain showing type IIx fibres. White bar = 30 µm

**Fig. 7:**
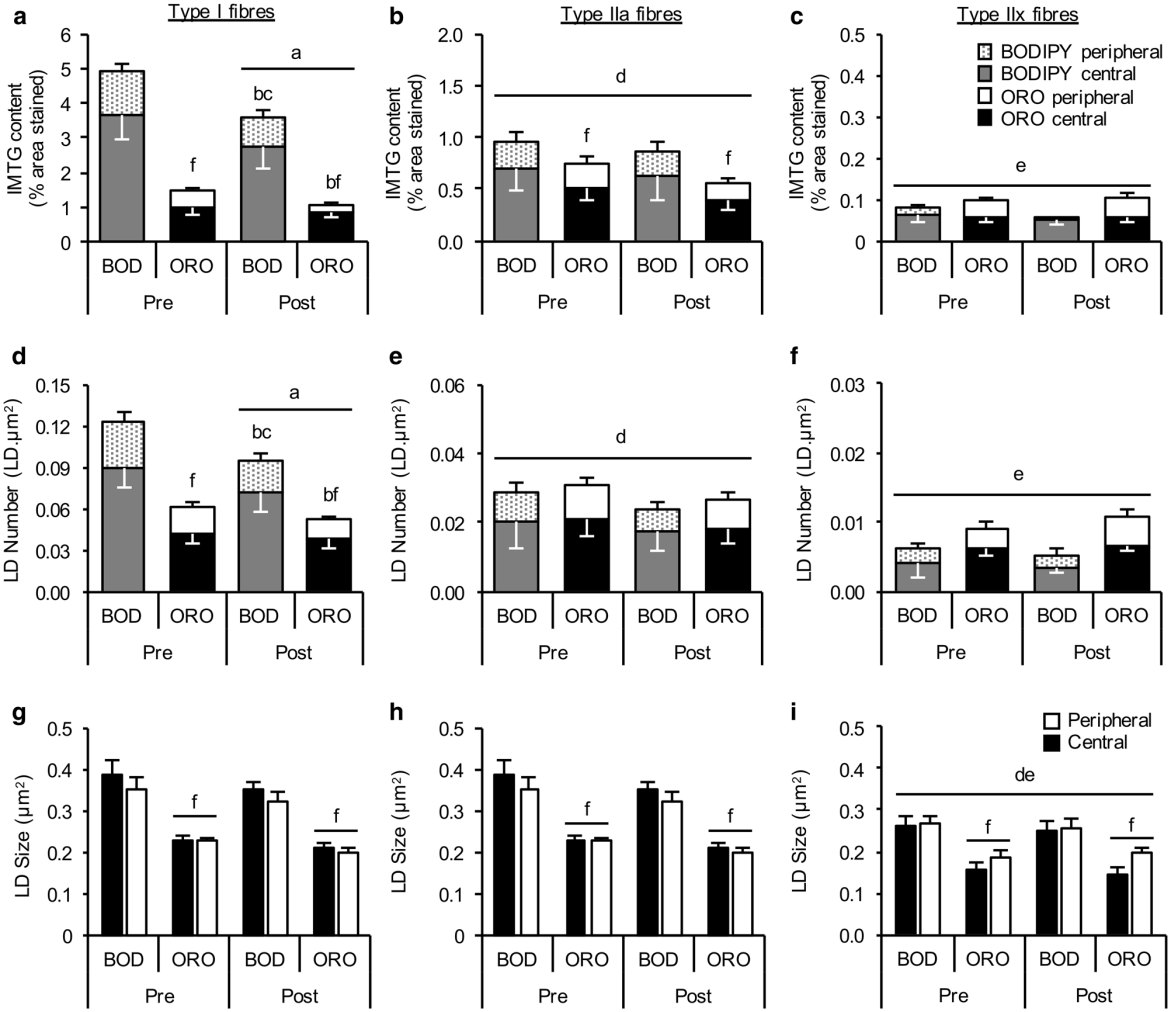
Region-specific IMTG use during exercise labelling LDs with BODIPY and ORO. IMTG content (**a**–**c**), LD number (**d**–**f**) and LD size (**g**–**i**) in peripheral and central subcellular regions in type I, type IIa and type IIx fibres, respectively, stained using BODIPY and ORO. IMTG content and LD number in each region was normalized to total cell area. ^a^Different from pre-exercise (*P *< 0.05), ^b^Difference in peripheral region from pre to post-exercise (*P *< 0.05); ^c^Difference in central region from pre to post-exercise (*P *< 0.05); ^d^Different from type I fibres (*P *< 0.05); ^e^Different from type IIa fibres (*P *< 0.05); ^f^Different from corresponding BOD value (*P *< 0.05). Values are mean ± S.D

Irrespective of the stain used, IMTG content was reduced in type I fibres only in response to 60 min moderate-intensity exercise (fibre × time interaction; *P *= 0.048 (Figs. 6, 7a). No changes in IMTG content were observed following exercise in type IIa (*P *= 0.15) or IIx fibres (*P *= 0.63). Based on this initial observation, we next compared exercise-induced changes in IMTG content between stains on a region-specific basis within each fibre type (Fig. 7a). To this end, using BODIPY we observed a reduction in IMTG content in both the peripheral (*P *= 0.003) and central regions (*P *= 0.044) of type I fibres in response to 60 min moderate-intensity exercise. However, using ORO only a significant decrease in IMTG content in the peripheral region of type I fibres was observed (*P *= 0.007), with no change detected in the central region (*P *= 0.18; Fig. 7a). Moreover, irrespective of the stain used, the region-specific reductions in IMTG content in type I fibres were attributed to a reduction in LD number (*P *= 0.003) (Fig. 7d), but not LD size (*P *= 0.028) (Fig. 7g).

## Discussion

Immunofluorescence microscopy techniques used to visualise and quantify IMTG content in human skeletal muscle have informed our current understanding of fibre type-specific IMTG utilisation during exercise, as well as the relationship between IMTG content and insulin resistance. These observations are largely based on the results of studies that have used ORO to visualise and quantify IMTG content in human skeletal muscle. However, there are several limitations to consider when using ORO. For example, ORO has a large emission spectrum (576-750 nm) which results in a high background level of staining, and consequently it is often difficult to distinguish between the background staining and a positive lipid stain. Furthermore, ORO stains all neutral lipids, including membrane phospholipids (Prats et al. 2013) and cholesterol esters (Kruth and Fry 1984). Here, we aimed to directly compare ORO to another lipid dye, BODIPY, which is becoming increasingly popular as a means to detect and quantify IMTG content in human skeletal muscle.

The first novel finding was that using the lipid dye BODIPY resulted in a higher estimate of IMTG content compared to ORO in all fibre types of human skeletal muscle. Moreover, this was driven by BODIPY detecting a greater number of LDs that, on average, were larger in size. This may seem counter-intuitive, because one might assume that a higher background staining would actually increase the size of objects that are considered to be positive staining. However, by employing stringent positive detection limits for roundness and intensity, this can be avoided. Consequently, the probability of detecting smaller, non-specific objects may actually be increased when using ORO. In contrast, BODIPY can be imaged with minimal background staining present, making it much simpler to distinguish positive lipid staining. As a result, the number of LDs that can be detected using BODIPY is increased.

Our second novel observation is that the LD detection properties for BODIPY and ORO appear to diverge by subcellular region. That is, IMTG content detected using BODIPY was greater in the central region compared to the peripheral region (5 µm beneath membrane), whereas IMTG content detected using ORO was not different in the peripheral region compared to the central region. Given that ORO is not solely specific to IMTG it is possible that the greater proportion of IMTG detected by ORO staining in the peripheral region is due to the detection of membrane lipids (Prats et al. 2013) such as cholesterol esters (Kruth and Fry 1984). This highlights why the specificity of the dye of choice is important. In contrast, BODIPY is considered to be more specific to IMTG, at least when imaged at 570 to 650 nm, with minimal staining of membrane lipids (Prats et al. 2013). Therefore, the observation that IMTG content detected using BODIPY was greater in the central region compared to the peripheral region could be considered to more accurately reflect subcellular differences in IMTG content. In this regard, studies employing transmission electron microscopy to investigate subcellular lipid content support this assertion, because they also demonstrate IMTG content to be greater in the central (or intermyofibrillar) region (Koh et al. 2017). Thus, it appears that the choice of lipid dye will have implications for the conclusions that are drawn from studies investigating regional differences in IMTG content and LD morphology in human skeletal muscle in relation to health and disease.

As with any laboratory assay, the quality of the data it can generate is dependent on the variation of the assay, and we considered this for both ORO and BODIPY. We report that BODIPY has a CV of ~ 6% vs. ~ 9% for ORO (at least in our hands), demonstrating that labelling of LD with BODIPY is less variable than using ORO. We also show that BODIPY is more photo-stable compared to ORO, at least when measuring IMTG content. This is important from a methodological perspective as image capture may not occur immediately on completion of staining procedure. We imaged the same fibres over a period of 13 days during which time the BODIPY staining was more photo-stable with 32% decrease in signal *vs.* 63% for ORO (Fig. 4). We do acknowledge that it would be both unusual and not recommended for stained slides to ever be imaged for the first time after 13 days. Nevertheless, the rate of the decline of the signals should perhaps be considered when using these immunofluorescence methods as both BODIPY and ORO showed a similar decrease in IMTG content after 3 days; however, at 6 days post-staining, there was decrease in lipid content detected of ~ 8% with BODIPY but ~ 19% with ORO and this decline continued to diverge until day 13. When considering LD morphology, we observed changes in both LD number and size that were dependent on the dye used 3 days after staining (Fig. 4). Thus, divergence in LD morphology (from day 1) occurred much sooner than significant changes in estimates of IMTG content, using either BODIPY or ORO. Taken together, this clearly has implications for the validity of the data generated and we suggest that the rate of signal decline, as well as the outcome of interest (i.e. content vs. morphology-related measures) is considered when conducting immunofluorescence microscopy experiments, especially when signal intensity is a key quantifiable outcome measure.

The data in the current study were generated from the analysis of 20 type I and 20 type IIa cells, which included ~ 12,000 LDs per participant for BODIPY analysis and ~ 5000 LDs per participant for ORO analysis. This is a much larger number of LDs analysed than has been reported in previous work using similar analytical methods and in a similar population (e.g., ~ 4000 LDs analysed by Gemmink et al. 2016). Thus, 20 type I fibres and 15 type IIa fibres could be considered exhaustive, as well as time-consuming to image and analyse. Therefore, as part of the current investigation we aimed to determine an appropriate number of fibres to be imaged in future analyses. This was completed by comparing the variation from the mean (of 20 or 15 type I or type IIa fibres, respectively) when cumulatively analysing more fibres (from 1 fibre up to 15 or 20 fibres). Based on our relatively small sample of 6 individuals, this analysis revealed that 13 cells per fibre type results in an ~ 10% variation from the mean (of 20 fibres). Since we report a coefficient of variation for our assays of < 10% (for both BODIPY and ORO), and most acute and chronic exercise interventions will induce changes in IMTG content that exceed 10% (Gemmink et al. 2016; Shepherd et al. 2012, 2013, Shepherd et al. 2017; van Loon et al. 2003), analysis of at least 13 fibres per fibre type (per time point) should provide the opportunity to detect a true and meaningful change in IMTG content. Whilst we do acknowledge that this number of fibres is unique to our data, future studies may use this as a point of reference, and should combine this information with their own CV for the assays that they use to determine changes in IMTG content.

Using the optimised protocol and analytical procedure, we compared BODIPY and ORO for determining fibre type and subcellular-specific IMTG utilisation during exercise. Irrespective of the lipid dye used, IMTG utilisation during exercise was specific to type I fibres, in line with a number of previous observations (Jevons et al. 2020; Shepherd et al. 2013; van Loon et al. 2003). However, the choice of lipid dye did reveal differences in IMTG utilisation during exercise when investigated on a subcellular-specific basis. While IMTG utilisation was of a similar magnitude in the central and peripheral regions when using BODIPY, utilisation of IMTG was specific to the peripheral region when examined using ORO. This latter finding is in direct contrast with a recent study employing transmission electron microscopy which reported that IMTG utilisation during high-intensity exercise occurred exclusively in the intermyofibrillar region (Koh et al. 2017). Whilst we acknowledge that using BODIPY may also not entirely replicate the observations made using high-resolution electron microscopy (because we observed exercise-induced decreases in IMTG content in both subcellular regions), our data suggest subcellular-specific IMTG utilisation during exercise should not be investigated using ORO. Future work that uses BODIPY to detect IMTG should likely employ a smaller peripheral region (e.g., 2 µm as used in Jevons et al. 2020) since this may be more representative of the subsarcolemmal region detected using high-resolution electron microscopy.

In summary, our investigation has highlighted important differences between BODIPY and ORO for detecting and quantifying IMTG on a fibre type and subcellular-specific basis. We propose that BODIPY should be the dye of choice to investigate IMTG in skeletal muscle due to its lower CV, greater photo-stability and capability to specifically detect IMTG avoiding quantification of membrane structures, compared to ORO.
